# Effect of sex chromosomes versus hormones in neonatal lung injury

**DOI:** 10.1172/jci.insight.146863

**Published:** 2021-07-08

**Authors:** Sandra L. Grimm, Xiaoyu Dong, Yuhao Zhang, Alexandre F. Carisey, Arthur P. Arnold, Bhagavatula Moorthy, Cristian Coarfa, Krithika Lingappan

**Affiliations:** 1 Molecular and Cellular Biology Department,; 2 Center for Precision Environmental Health, and; 3 Department of Pediatrics, Texas Children’s Hospital, Baylor College of Medicine, Houston, Texas, USA.; 4 Integrative Biology and Physiology, University of California, Los Angeles, California, USA.; 5 Dan L Duncan Comprehensive Cancer Center, Baylor College of Medicine, Houston, Texas, USA.

**Keywords:** Pulmonology, Bioinformatics, Mouse models, Sex hormones

## Abstract

The main mechanisms underlying sexually dimorphic outcomes in neonatal lung injury are unknown. We tested the hypothesis that hormone- or sex chromosome–mediated mechanisms interact with hyperoxia exposure to impact injury and repair in the neonatal lung. To distinguish sex differences caused by gonadal hormones versus sex chromosome complement (XX versus XY), we used the Four Core Genotypes (FCG) mice and exposed them to hyperoxia (95% FiO_2_, P1–P4: saccular stage) or room air. This model generates XX and XY mice that each have either testes (with *Sry*, XXM, or XYM) or ovaries (without *Sry*, XXF, or XYF). Lung alveolarization and vascular development were more severely impacted in XYM and XYF compared with XXF and XXM mice. Cell cycle–related pathways were enriched in the gonadal or chromosomal females, while muscle-related pathways were enriched in the gonadal males, and immune-response–related pathways were enriched in chromosomal males. Female gene signatures showed a negative correlation with human patients who developed bronchopulmonary dysplasia (BPD) or needed oxygen therapy at 28 days. These results demonstrate that chromosomal sex — and not gonadal sex — impacted the response to neonatal hyperoxia exposure. The female sex chromosomal complement was protective and could mediate sex-specific differences in the neonatal lung injury.

## Graphical abstract

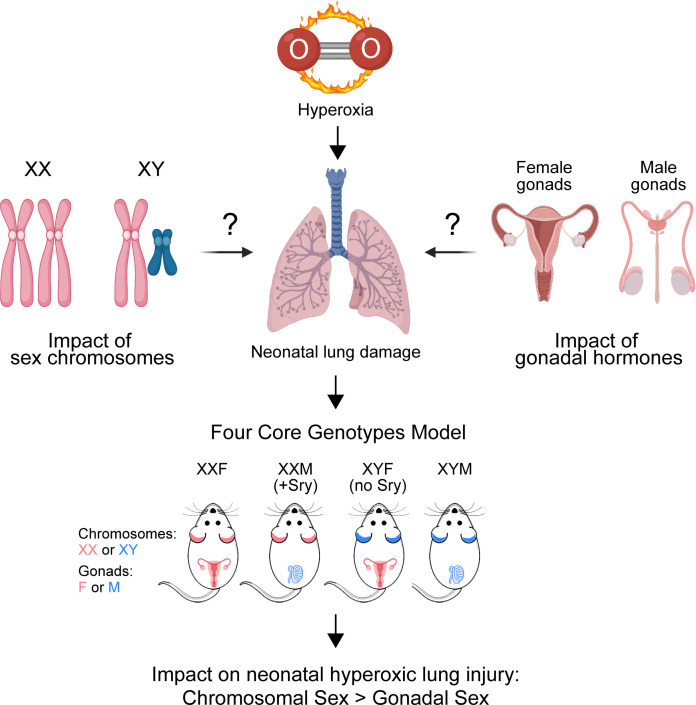

## Introduction

Sex as a biological variable plays a crucial role both during lung development and in modulating recovery from neonatal lung injury. Bronchopulmonary dysplasia (BPD), a lung disease characterized by aberrant alveolar and pulmonary vascular development, causes significant morbidity in preterm neonates. The incidence of this disease is skewed toward the male sex (1). In a recently published study from the Canadian Neonatal Network, among neonates born at < 29 weeks’ gestation between 2007 and 2016, the composite of death or major morbidity was higher in males over the study period and the difference between rates of the major morbidities including BPD was unchanged between male and female premature neonates (2). The sex differences have been replicated in murine models and are strain specific (3, 4). Male mice have greater alveolar simplification, impaired pulmonary vascular development, and more long-term adverse sequelae compared with female mice (5, 6).

**Figure 1:**
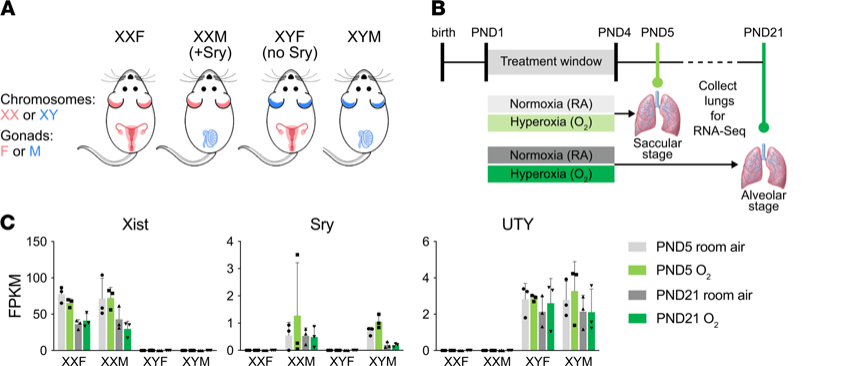
The FCG mouse model, neonatal hyperoxia exposure, and whole lung RNA-Seq experimental model. (**A**) Mice from the FCG model. XXF (chromosomally and gonadally female), XXM (chromosomally female, gonadally male), XYF (chromosomally male, gonadally female), and XYM (chromosomally and gonadal male) were exposed to hyperoxia (95% FiO_2_ from P1–P4) during the saccular stage of lung development and euthanized on P5 and P21. (**B**) Whole lung mRNA was subjected to RNA-Seq analysis (*n =* 3/group). (**C**) Genotyping was confirmed by the FPKM levels for *Uty, Sry*, and *Xist*. Data are shown as mean ± SD.

The underlying molecular mechanisms behind these sex-specific differences may range from effects of sex chromosomes versus the differential exposure to sex hormones prenatally (7). To distinguish sex differences caused by gonadal hormones versus sex chromosome complement (XX versus XY), we used the Four Core Genotypes mice (FCG) (8). In the FCG model (Figure 1), *Sry* is a transgene and is not present on the Y chromosome, meaning that XX and XY mice can each have either testes (with *Sry*, XXM, or XYM) or ovaries (without *Sry*, XXF, or XYF). The model also has an advantage in that it tests simultaneously for hormonal effects and the interaction of sex chromosome and hormonal effects. The XYM male (gonadal and chromosomal male) in this model bears the *Sry* gene on an autosome instead of the Y chromosome. When a WT XXF female is bred with the XYM male, the pups could be any of the 4 genotypes: XXM (gonadal male with XX chromosomal complement), XYM (gonadal male with XY chromosomal complement), XXF (gonadal female with XX chromosomal complement), or XYF (gonadal female with XY chromosomal complement). Our objective was to test the hypothesis that hormonal or sex chromosome mechanisms interact in the sex-specific modulation of neonatal hyperoxic lung injury. We assessed changes in lung alveolarization and pulmonary vascular development after neonatal hyperoxia exposure (during the saccular stage of lung development P1–P4) in the FCG mice. In addition, differences in the pulmonary transcriptome and the underlying biological pathways and regulatory networks were elucidated and compared with changes in human patient gene expression stratified by sex (Figure 1).

## Results

### Chromosomal sex had a significant impact on alveolarization after neonatal hyperoxia exposure and not gonadal sex.

**Figure 2:**
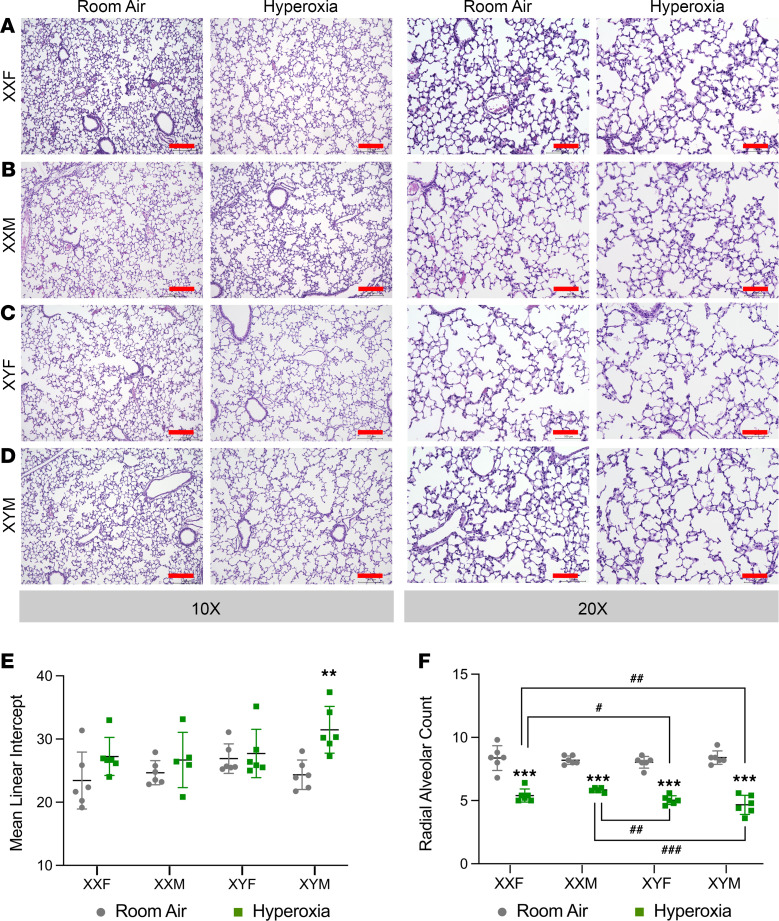
Chromosomal sex but not gonadal sex had a significant impact on alveolarization after neonatal hyperoxia exposure in mice. (**A**–**D**) Representative H&E-stained sections from male and female neonatal mice exposed to room air or hyperoxia (95% FiO_2_, P1–P4) at ×10 and ×20 magnification in XXF (**A**), XXM (**B**), XYF (**C**), and XYM (**D**) mice. Lung morphometry in neonatal FCG mice (*n =* 5–6 mice per group) exposed to hyperoxia (95% FiO_2_, P1–P4) was assessed using mean linear intercept (MLI) and radial alveolar count (RAC). (**E**) MLI in FCG neonatal mice exposed to room air or hyperoxia on P21. (**F**) RAC in FCG neonatal mice exposed to room air or hyperoxia on P21. Data are shown as mean ± SD from 5–6 individual animals. Statistical analysis was performed using 3-way ANOVA to assess the effect of treatment, chromosomal sex and gonadal sex, as well as the interactions between the independent variables. Significant differences between room air and hyperoxia within genotype are indicated by ***P <0.001. Significant differences between hyperoxia-exposed mice between different genotypes are indicated by ^#^*P* < 0.05, ^##^*P* < 0.01, and ^###^*P* < 0.001. Scale bars: 200 μm (×10) or 100 μm (×20).

Representative lung sections (P21) stained with H&E from the FCG mice (room air and hyperoxia-exposed) are shown in Figure 2, A–D. Impact on lung alveolarization was measured using lung morphometric indices. The mean linear intercept (MLI) and radial alveolar count (RAC) were not different between the 4 genotypes at baseline under room air conditions (Figure 2, E and F). Statistical analysis by 3-way ANOVA showed a main effect of treatment and chromosomal sex, but not gonadal sex. Among the interactions, the interaction between chromosomal sex and treatment was statistically significant for RAC. The interaction between all 3 factors (gonadal sex, treatment, and chromosomal sex) was also significant. Compared with room air controls, the MLI was significantly increased in XYM mice. The RAC was decreased in all 4 genotypes upon exposure to hyperoxia. Compared with XYM and XYF mice, the XXM and the XXF mice had better preservation of the RAC upon exposure to hyperoxia. The detailed report of 3-ANOVA is shown in Supplemental Table 1 (supplemental material available online with this article; https://doi.org/10.1172/jci.insight.146863DS1).

### Arrest in angiogenesis after hyperoxia exposure is significantly impacted by chromosomal sex but not gonadal sex.

**Figure 3:**
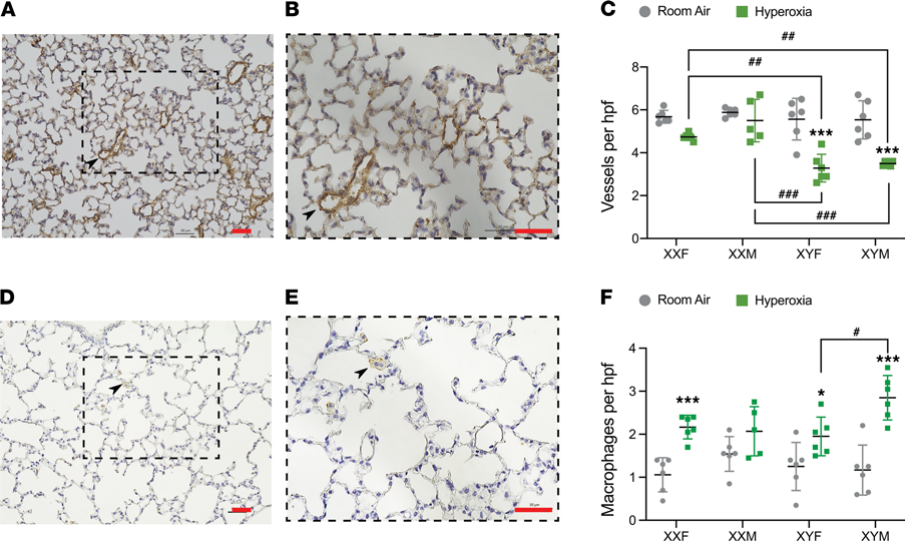
Arrest in angiogenesis and lung macrophages after hyperoxia exposure is differentially impacted by chromosomal and gonadal sex in neonatal mice. IHC and quantitation of pulmonary microvessels was done by immunostaining for endothelial-specific vWF (*n =* 5–6 mice per group) in room air or hyperoxia (95% FiO_2_, P1–P4) in XXF, XXM, XYF, and XYM mice at P21. (**A** and **B**) Representative stained sections at ×20 (**A**) and ×40 magnification (**B**). Arrows point to brown-staining vessels. (**C**) Quantitative analyses showing number of vessels per high-power field (×20) in lungs of FCG neonatal mice. IHC and quantitation of macrophages was done by immunostaining for F4/80 (*n =* 5–6 mice per group) in room air or hyperoxia (95% FiO_2_, P1–P4) in XXF, XXM, XYF, and XYM mice at P21. (**D** and **E**) Representative stained sections at ×20 (**D**) and ×40 magnification (**E**). Arrows point to brown-staining macrophages. (**F**) Quantitative analyses showing number of macrophages per high-power field (×20) in lungs of FCG neonatal mice. Data are shown as mean ± SD from 5–6 individual animals. Statistical analysis was performed using 3-way ANOVA to assess the effect of treatment, chromosomal sex, and gonadal sex, as well as the interactions between the independent variables. Significant differences between room air and hyperoxia within genotype are indicated by *P <0.05 and ***P <0.001. Significant differences between hyperoxia-exposed mice between different genotypes are indicated by ^##^*P* < 0.01 and ^###^*P* < 0.001. Scale bars: 50 μm.

Pulmonary vessel density was determined based on von Willebrand Factor (vWF) immunostaining, which is an endothelial-specific marker. vWF-stained vessels with external diameter (<50 μm per high-power field) were quantitated. A representative lung section stained with vWF at P21 is shown in Figure 3, A and B, with quantitation for all genotypes represented in Figure 3C. Statistical analysis by 3-way ANOVA showed a main effect of treatment and chromosomal sex but not gonadal sex. The interaction between chromosomal sex and treatment was statistically significant. Compared with room air controls, the vessel count was significantly decreased in XYM and XYF mice. Interestingly, the hyperoxia-exposed XXM and XXF mice were protected and had a higher vessel count compared with either the XYM or the XYF mice. We also performed IHC for macrophages and quantitation of the same at P21 (Figure 3, D, E, and F). Interestingly, we found — compared with room air controls — macrophages were increased in the hyperoxia-exposed lungs in XXF, XYM, and XYF mice. Furthermore, the increase in XYM mice was greater compared with XYF mice. Statistical analysis by 3-way ANOVA showed a significant effect of treatment and gonadal sex. The interaction between all 3 factors (gonadal sex, treatment, and chromosomal sex) was also significant. The detailed report of 3-ANOVA is shown in Supplemental Table 1.

### Differences in the lung transcriptome in the FCG model after neonatal hyperoxia exposure.

**Figure 4:**
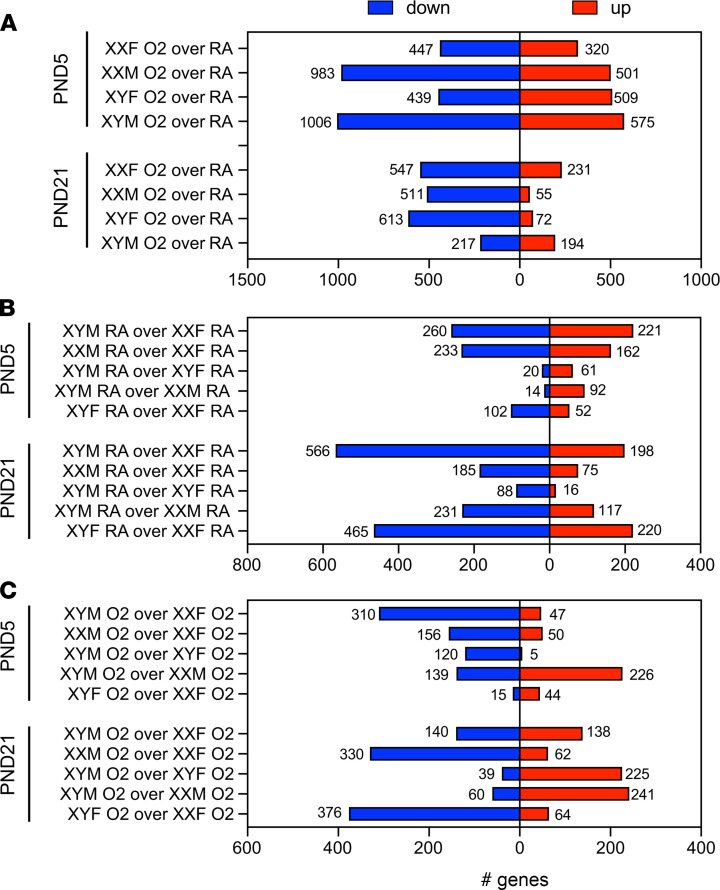
Transcriptomic analysis of FCG mice shows a robust and genotype-specific response to neonatal hyperoxia exposure. The total number of up- and downregulated differentially expressed genes (DEGs). (**A**–**C**) The hyperoxia response in each genotype is shown in **A**, while differences between the genotypes in normoxia and under hyperoxic conditions are shown in **C** and **B**, respectively.

The FCG mouse lung transcriptomes were profiled using RNA sequencing (RNA-Seq). The total number of up- and downregulated differentially expressed genes (DEGs) in individual comparisons are shown in Figure 4. The hyperoxia responses (hyperoxia [O_2_] versus normoxia [RA]) in each genotype are shown in Figure 4A, while differences between the genotypes in normoxia and under hyperoxic conditions are shown in Figure 4, B and C, respectively. In Figure 4A, at P5, there are more downregulated than upregulated genes in all the genotypes except XYF mice. Interestingly, at P21, the XYM mice had almost an equal number of up- and downregulated genes, whereas the other genotypes continued to have a predominance of downregulated genes. Genes on the X and Y chromosome were also analyzed. There were no genes on the Y chromosome that were differentially expressed in response to hyperoxia in the neonatal lung at P5 or at P21. There were 12 upregulated X-genes in XXF and 20 upregulated X-genes in XXM at P5. At P21, 8 X-genes were upregulated in XXF and 1 gene in XXM. One of the common X-chromosome genes that was upregulated in XXM and XXF was apelin (*Apln*). Apelin is highly expressed in the vascular endothelium and attenuates hyperoxic lung and heart injury in neonatal rats (9). Four Y-chromosome genes (*Eif2s3y, Kdm5d, Ddx3y,* and *Uty)* were expressed to a greater extent in XY mice compared with XX mice both under room air and hyperoxia at both the time points. There were many X-chromosome–specific genes that were higher in XX mice under normoxia at P5, and this trend could have biased the response to hyperoxia in these mice. These genes included *Kdm5c* (XXF > XYM) and *Kdm6a* (XXM > XYM)*,* which are both histone lysine demethylases and can, thus, modulate the epigenome and have been implicated in sexually dimorphic diseases (10). *Kdm6a* is a direct sensor of oxygen and controls chromatin and cell fate (11). *Kdm5c* has recently been implicated in sex-specific differences in adiposity (12) and, interestingly, is expressed to a greater extent in female HUVECs compared with male (13). The complete list of DEGs in each genotype at different time points, as well as the X- and Y-chromosome–specific genes, is shown in Supplemental Table 2. The comparison of gene expression between chromosomally male and chromosomally female mice stratified for X-chromosome–associated and autosomal genes on the 4 core genotypes mice under normoxic and hyperoxic conditions at P5 and 21 is shown in Supplemental Figure 1.

### Clustering of the FCG murine transcriptome responses based on chromosomal and gonadal patterns.

**Figure 5:**
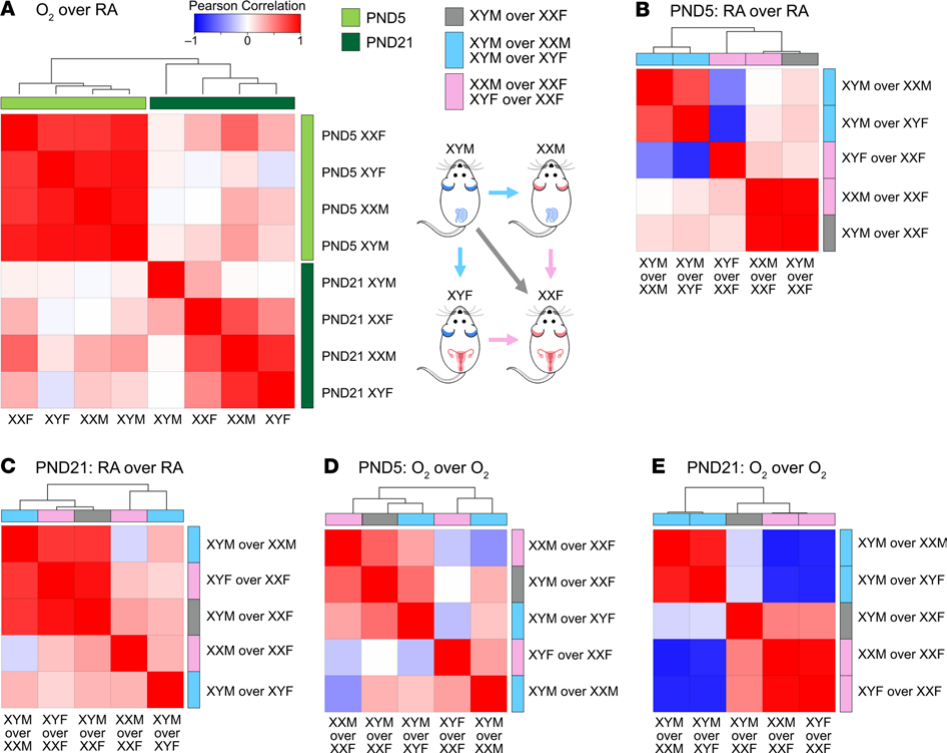
Gene expression signatures in the FCG murine neonatal lungs show striking correlation patterns superimposed on the human lung transcriptome. To compare signatures (differentially expressed genes; DEGs) in our pulmonary murine models, we used transcriptome profiles of 578 healthy human adult lung samples compiled by the GTEx consortium to assess genotype/transcriptome relationships in phenotypically healthy individuals. We computed summed *Z*-scores for each human individual and each FCG signature and assessed intersignature correlations. (**A**) There is a clear separation between the P5 and the P21 hyperoxia signatures. The acute response signatures at P5 show very strong correlation across all genotypes. At P21, the hyperoxia responses in gonadal or chromosomal females XXF, XXM, and XYF cluster together, apart from the response in the chromosomal and gonadal male XYM genotype. (**B**–**E**) Correlation between genotype summed *Z*-scores are shown in room air at P5 (**B**) and P21 (**C**), and hyperoxia at P5 (**D**) and P21 (**E**).

**Figure 6:**
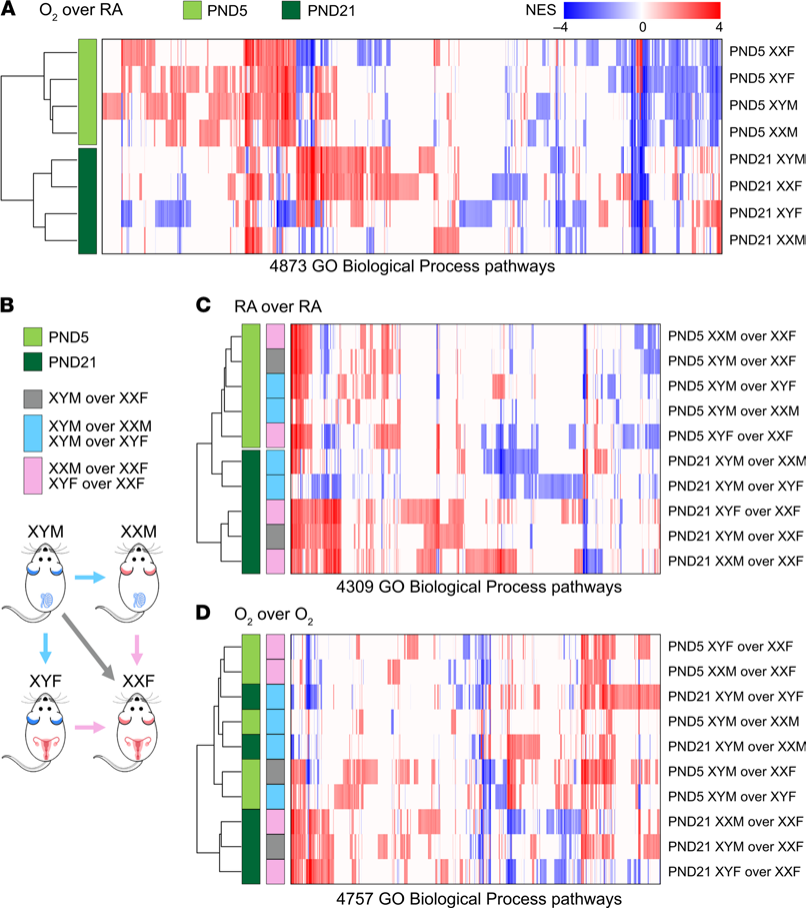
Pathway-based cluster analysis for transcriptomic footprints of hyperoxia exposure or of genotype differences in the FCG mouse model. Gene Set Enrichment Analysis (GSEA) was used to quantify enrichment of GO Biological Process pathways. Hierarchical clustering and heatmaps were generated for the transcriptomic footprints using the significant normalized enrichment scores (NES). (**A**) Pathway-based clustering of response to hyperoxia across all genotypes show striking and distinct clustering between the P5 and P21 responses. (**B**) Overview of intergenotype comparisons. (**C**) Pathway-based clustering of intergenotype differences under room air conditions also show robust clustering with the time points. (**D**) Hierarchical clustering of enriched biological pathways under hyperoxia of intergenotype comparisons does not show clustering with respect to post-natal day.

To compare signatures (DEGs) in our pulmonary murine models, we used transcriptome profiles of 578 healthy human adult lung samples compiled by the GTEx consortium, one of the largest-scale efforts to assess genotype/transcriptome relationships in phenotypically healthy individuals (14). We computed summed *Z*-scores for each human individual and each FCG signature and assessed intersignature correlations, as described in the Data Supplement. There is a clear separation between the P5 and the P21 hyperoxia signatures, as shown in Figure 5A. The acute response signatures at P5 show very strong correlation across all genotypes. Scatterplots presenting summed *Z*-scores at individual specimen level, irrespective of biological sex, for all pairs of P5 hyperoxia responses are shown in Supplemental Figure 2A. At P21, the hyperoxia responses in gonadal or chromosomal females XXF, XXM, and XYF cluster together, separately from the XYM genotype. This pattern persisted, despite separation of the human cohort into male and female samples, as shown in Supplemental Figure 2, B and C. Correlation of genotype summed *Z*-scores in room air at P5 show that signatures of comparisons between gonadal males and chromosomal males are highly correlated (Figure 5B). At P21, however, signatures of male chromosomes over female chromosomes are highly correlated, as are signatures of male gonadal sex over female gonadal sex with the same sex chromosomal complement (Figure 5C). Next, correlation between genotype transcriptomic footprint in hyperoxia at P5 indicated that all 3 signatures of male gonads over female gonads are highly correlated (Figure 5D). Finally, at P21 there was a striking distinction with signatures of gonadal and chromosomal males clustering together as opposed to gonadal and chromosomal females (Figure 5E). For another perspective of the gene expression changes, we employed pathway-based cluster analysis for transcriptomic footprints of hyperoxia exposure or of genotype differences in the FCG model. Gene Set Enrichment Analysis (GSEA) was utilized to determine enrichment of Gene Ontology (GO) Biological Process pathways. Each transcriptomic response was represented based only on the normalized enrichment scores (NES) of significantly enriched pathways (FDR < 0.25); then, hierarchical clustering was used to determine relationships between transcriptome responses. Pathway-based clustering of hyperoxia response across all FCG genotypes show clear clustering between the 2 time points P5 (end of saccular stage of lung development) and P21 (alveolar stage of lung development) (Figure 6A). Interestingly, while there are small differences according to sex variables in footprints at P5, there was a fairly striking interaction of sex chromosomes and gonads at P21. Similarly, pathway-based clustering of intergenotype differences (outlined in Figure 6B) in room air conditions also showed a clear separation of the time points, pointing toward a temporality in gene expression in the developing lung (Figure 6C). Enriched biological pathways in intergenotypes comparisons in hyperoxia show a disruption of the temporal patterns observed in room air (Figure 6D).

### Effect of chromosomal and gonadal sex on gene expression in the developing lung in response to hyperoxia exposure.

**Figure 7:**
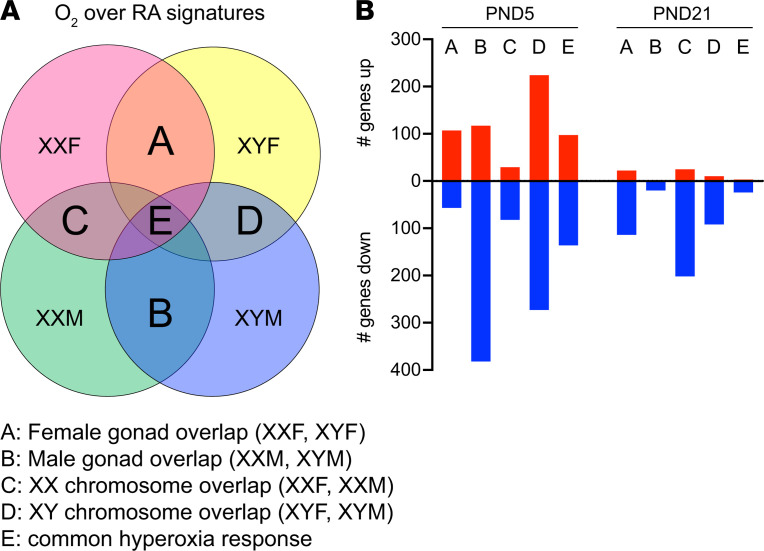
Distribution and overlap of gene signatures in the FCG mouse model based on the gonadal and chromosomal sex. (**A**) The 4-way Venn diagram shows the schematic used to discern the number of genes (up- and downregulated) based on female gonadal response (area A; common genes between XXF and XYF), male gonadal response (area B; common genes between XXM and XYM), female chromosomal response (area C; common genes between XXF and XXM), and male chromosomal response (area D; common genes between XYF and XYM). Area E (which represents the common differentially expressed genes [DEGs] across all the genotypes) was not included for the groups above. (**B**) The adjoining graph provides the number of genes from each of these groups.

We wanted to elucidate the DEGs in response to hyperoxia exposure based on the chromosomal and gonadal sex (Figure 7). The genes in region A of Figure 7 depict the common DEGs between XXF and XYF (gonadal females), in region B depict the common DEGs between XXM and XYM (gonadal males), in region C include XXF and XXM (chromosomal female), and in region D include XYF and XYM (chromosomal male). These overlaps do not include the region E, which comprises the common subset of DEGs that were present in all the 4 genotypes (Figure 7A). Several interesting patterns emerge in this analysis. At P5, the gonadal males and the chromosomal males have the greatest number of DEGs. In addition, with the exception of the female gonadal response, which had 65.2% upregulated genes, all the other responses were composed predominantly of downregulated genes. In total, 76.5% of DEGs in the male gonadal response and 73.9% of DEGs in the female chromosomal response were downregulated. The male chromosomal response was more evenly distributed between up- and downregulated genes. At P21, there was a predominant shift to mainly downregulated genes in all responses. In contrast, with the previous time point, the female gonadal and chromosomal response had the greatest number of DEGs. There were no upregulated genes in the male gonadal response. A detailed report of the number of DEGs in each genotype and the overlap based on gonadal or chromosomal sex is shown in Supplemental Figure 4. The overlap immediately after hyperoxia exposure was the highest between XYM and XYF mice, with 40% of DEGs being common. The overlap was the least in the chromosomal female (XXM and XXF) mice at 18% and was about 30% in gonadal females and males. This overlap decreased in most genotypes at P21, except in chromosomal females (XXM and XXF), where it increased to 24% from 18%. The overlap was least in gonadal males (between XYM and XXM) and was around 13% in gonadal females (XYF and XXF) and chromosomal males (XYM and XYF). In the common hyperoxia response in region E, there were 233 DEGs at P5 (97 upregulated, 136 downregulated) and 27 DEGs at P21. A detailed list of these genes is included in Supplemental Table 2.

### Pathway analysis of DEGs.

**Figure 8:**
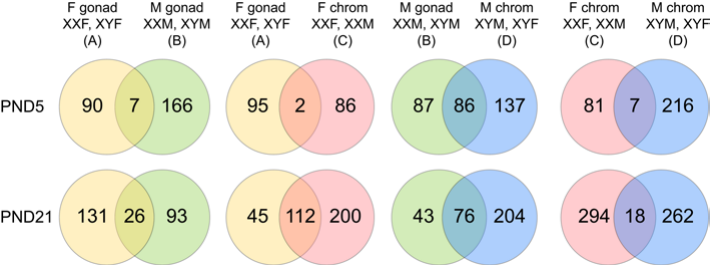
Distribution and overlap of biological pathways based on the gonadal and chromosomal sex in the FCG mouse model. Analysis of enriched pathway from the Gene Ontology Biological Processes compendium in DEGs and the overlap based on the gonadal or chromosomal sex at P5 and P21. The number of biological pathways are specified within the Venn diagrams.

**Figure 9:**
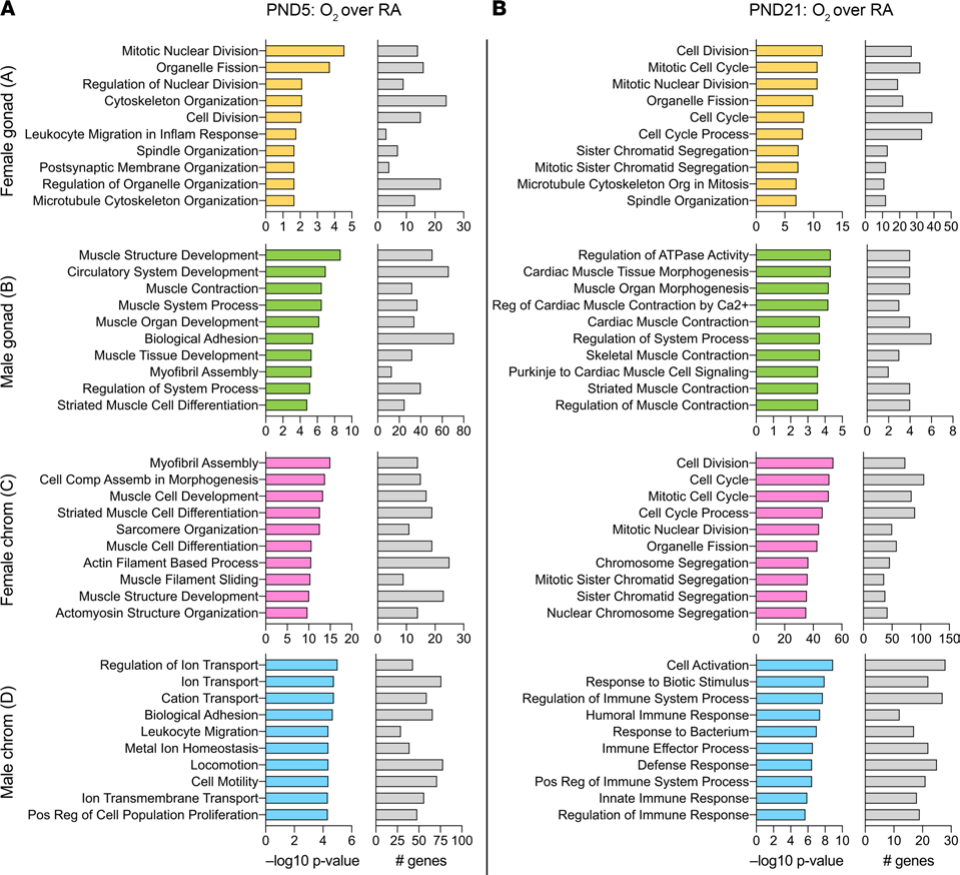
Enriched biological pathways are distinct based on the gonadal or chromosomal sex in the FCG mouse model at P5 and P21 after hyperoxia exposure. (**A** and **B**) The top 10 Gene Ontology Biological Process pathways enriched in mice with female gonads (area A; XXF and XYF), male gonads (area B; XXM and XYM), female chromosomes (area C; XXF and XXM), and male chromosomes (area D; XYM and XYF) are shown. The significance of enriched pathways as –log_10_ (*P* value) and the number of included DEGs in the pathways are depicted.

To identify biological processes that were enriched in gene signatures of FCG mice after exposure to hyperoxia, we used the GO Biological Processes compendium. The number of enriched pathways and the overlap between gonadal and chromosomal sex is shown in Figure 8. At P5, the highest overlap was seen between mice with male gonads (XXM and XYM) and male chromosomes (XYM and XYF) with 27.7% overlap. At P21, however, the greatest overlap was seen between female gonads and female chromosomes, with 31.3% overlap. The other comparisons showed more common pathways at P21 compared with P5. The top 10 biological pathways enriched by gonadal or chromosomal sex are listed in Figure 9, along with their significance values and the number of included DEGs. At P5, cell division and cytoskeletal organization pathways were enriched in mice with female gonadal sex (XXF and XYF), while in mice with female chromosomal sex (XXM and XXF) and male gonadal sex (XYM and XXM), almost all enriched pathways were related to muscle cell development or differentiation. Circulatory system development was one of the top pathways in XXM and XYM mice. In mice with male chromosomes (XYM and XYF), ion transport, cell motility, and biological adhesion were among the top enriched pathways.

At P21, mice with female gonadal sex (XYF and XXF) and female chromosomal sex (XXF and XXM) were both enriched in pathways related to cell cycle and cell division. Pathways related to muscle contraction and muscle morphogenesis were enriched in mice with male gonadal sex. Interestingly, in mice with male chromosomal sex, immune response–related pathways predominated. Gene lists related to enriched pathways for all these comparisons are shown in Supplemental Table 3.

We further focused on pathways related to immune/inflammatory processes or lung development in this model (Supplemental Figure 3). As far as immune and inflammatory pathways are concerned, they were enriched in XXF mice at P5 and in XXM mice at P21 (Figure E3B). Interestingly, at P21, the XXM mice have unique enrichment of these pathways compared with the other 3 genotypes. Many of these pathways are related to the innate immune response and inflammatory response to antigenic stimulus. Lung alveolus and epithelium development is enriched in XXF mice at P5. At P21, XYF mice show greater enrichment of lung development–related pathways compared with the other 3 genotypes. Details of these pathways are provided in Supplemental Table 3.

### Independent quantitative PCR validation.

**Figure 10:**
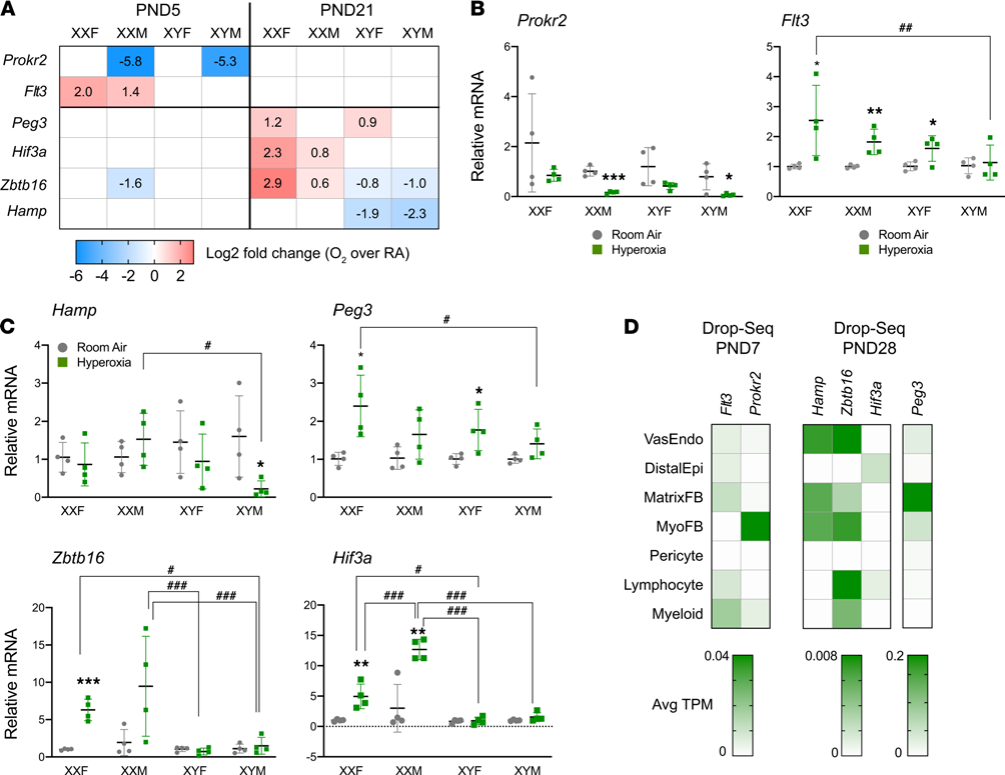
Independent validation of RNA-Seq results for selected genes with qPCR and cell-specific expression in neonatal mice. A subset of highly expressed differentially expressed genes were validated in an independent cohort of FCG neonatal mice. (**A**–**C**) The fold change of the selected genes in the RNA-Seq experiment are shown in **A**, and the changes in the qPCR experiments are shown for DEGs at P5 (*Flt3* and *Prokr2*) (**B**) and for DEGs at P21 (*Hamp, Peg3, Zbtb16 and Hif-3*α) (**C**). Data are shown as mean ± SD from 4 individual animals. Analysis done by 3-way ANOVA to assess the effect of treatment, chromosomal sex, and gonadal sex, as well as the interactions between the independent variables. Significant differences between room air and hyperoxia within genotype are indicated by *P < 0.05, **P < 0.01, and ***P <0.001. Significant differences between hyperoxia-exposed mice between different genotypes are indicated by ^#^*P* < 0.05, ^##^*P* < 0.01, and ^###^*P* < 0.001. (**D**) For these selected genes, we also evaluated the expression in publicly available neonatal lung single-cell expression database (LungMAP).

A subset of highly expressed differentially expressed genes were validated in an independent cohort of neonatal mice (Figure 10). The genes were selected based on the highest differential expression, either based on gonadal sex or chromosomal sex at either time point. The fold change of the selected genes in the RNA-Seq experiment are shown in Figure 10A, and the changes in the quantitative PCR (qPCR) experiments are shown in Figure 10B (P5) and Figure 10C (P21). The results and the known relevant biological significance are shown in Table 1. The detailed report of 3-way ANOVA is shown in Supplemental Table 1. Good correlation was seen for the validated genes between the RNA-Seq and the qPCR results. For these selected genes, we also evaluated the cell-specific expression using publicly available neonatal lung single-cell expression database (LungMAP) in Figure 10D. At P5, we validated *Flt3* (FMS-like tyrosine kinase 3) and *Prokr2* (prokineticin receptor 2). *Flt3* has the highest expression in myeloid cells and lymphocytes, and *Prokr2* has the highest expression in myofibroblasts in the murine lung at P7. At P21, we validated *Hamp* (hepcidin antimicrobial peptide), *Peg 3* (paternally expressed 3), *Zbtb16* (zinc finger and BTB domain containing 16), and *Hif-3*α. At the cellular level, Hamp has the highest expression in vascular endothelial cells, *Hif-3*α in the distal epithelium and the lymphocytes, *Peg 3* in matrix fibroblasts, and *Zbtb16* in endothelial cells and lymphocytes at P28 in the LungMAP data set. In addition, we validated genes related to inflammation/immune-related pathways including *Tlr7, Nr1d1* (nuclear receptor subfamily 1 group D member 1), *Alox15* (arachidonate 15-lipoxygenase), *Hk3* (hexokinase 3), *Rsad2* (radical s-adenosyl methionine domain containing 2), and *Ltf* (lactotransferrin) at P21. The summary of the genes and their known biological relevance is highlighted in the Data Supplement (Supplemental Table 1).

### Murine hyperoxia gene expression response associates with BPD blood transcriptomes.

**Figure 11:**
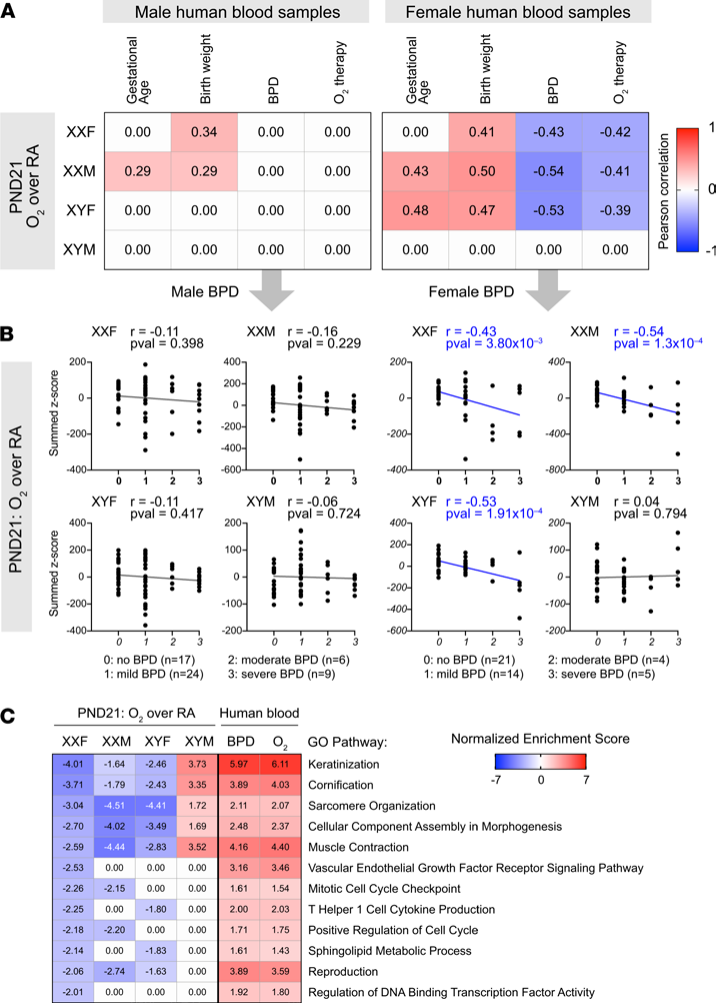
Murine hyperoxia signatures associate with blood transcriptomes in human newborns at risk of BPD. Blood transcriptome at P28 from a cohort of human newborns evaluated for development of BPD was obtained. (**A**) The distribution of summed *Z*-scores for hyperoxia gene signatures from all our murine models, at P21, was evaluated against 4 clinical variables: gestational age, birth weight, BPD status, and need for oxygen at 28 days of postnatal age. (**B**) Pearson correlation coefficient is shown for significant correlation (*P <* 0.05). Distribution of summed *Z*-scores for hyperoxia signatures in murine models at P21 are shown in the human newborn blood samples collected at P28, stratified by biological sex and by BPD status (no BPD, mild, moderate and severe). Association was evaluated using the parametric Pearson correlation, with the Pearson correlation coefficient (*r*) and *P* values indicated. (**C**) Selected Gene Ontology pathways and their normalized enrichment scores induced in the blood transcriptome of BPD patients at P28, while suppressed after hyperoxia exposure in lungs of feminized mice and either induced or not differentially modulated in XYM mice at P21, are shown in the heatmap.

A benefit of compiling transcriptomes in human clinical cohorts with rich clinical data is enabling a statistical evaluation of the association between individual gene signatures and clinical variables. We obtained blood transcriptome from a cohort of human newborns (15) evaluated for development of BPD, and we specifically analyzed the human transcriptomes at P28. We evaluated the distribution of summed *Z*-scores for the hyperoxia gene signatures from all our murine models at P21, over 4 clinical variables — gestational age, birth weight, BPD status, and oxygen requirement at 28 days — and we also stratified the human samples by sex. Interestingly, we saw striking patterns between murine hyperoxia signatures at P21 (Figure 11, A and B). The XXF and XXM chromosomal female signatures were correlated with birth weight in the male blood samples (Supplemental Figure 5). However, the chromosomal or gonadal female (XXF, XXM, and XYF) gene signatures were positively associated with birth weight and were anticorrelated with BPD and oxygen therapy at 28 days from the gene signatures from female patients. We next defined biological pathways that were upregulated in the human BPD patients at P28 but were suppressed after hyperoxia exposure in lungs of feminized mice and either induced or not differentially modulated in XYM mice at P21 (Figure 11C). Interestingly pathways related to keratinization and muscle contraction were among the top significantly enriched pathways that showed this pattern of modulation.

## Discussion

In this study, we show — for the first time to our knowledge — the impact of chromosomal sex and gonadal sex on neonatal hyperoxic lung injury and repair in mice. Chromosomal sex and interaction between chromosomal sex and treatment had a significant effect on both alveolarization and pulmonary vascular development in hyperoxia-exposed neonatal mice. Gonadal sex had a minor effect, as evidenced by a significant 3-way interaction between gonadal sex, chromosomal sex, and hyperoxia for lung development (Figure 2, E and F). XXM and XXF were protected against hyperoxic lung injury compared with neonatal mice with XYM and XYF. We also report the changes in the lung transcriptome and the distinct enriched biological pathways that program the lung recovery after early life injury.

The sexual dimorphism in neonatal outcomes has been highlighted in many clinical and basic science publications. The developing fetus is exposed to gonadal hormones during organogenesis, and many previous publications have reported on the deleterious effects of androgens on the developing lung (16). Conversely, the female sex hormones estrogen and progesterone are thought to have a protective effect (17, 18). However, after the immediate postnatal period, the developing lung is not exposed to high levels of circulating sex hormones; hence, hormone-independent effects could also underlie the sexual dimorphism seen in the neonatal lung. The FCG mouse model enables the separation of 2 main factors that underlie sex differences in disease pathophysiology: the sex chromosome complement (XX versus XY) and the gonadal hormones. Using this model enabled us to compare XX and XY mice that were exposed to a comparable hormonal environment (19). Itoh et al. showed that multiple copies of the *Sry* transgene are present in the FCG mice on chromosome 3 and that these mice do not show evidence of being exposed to different androgen levels prenatally (20). There were also no differences in postnatal gonadal hormonal levels between mice with a similar gonadal makeup (21).

The FCG model has been used to answer the question of whether a full understanding of the gonadal hormones or the sex chromosomal complement is required to understand sexual dimorphism in many diseases and processes, including autoimmune diseases, stroke, neurobehavioral derangements, nociception, and drug abuse (19, 21). Interestingly, in gonadectomized FCG mice, XY mice — irrespective of gonadal sex — developed less severe hypoxia-induced pulmonary hypertension than XX mice (22). In viral infections, the sex chromosomal complement played a role in the pathogenesis and outcomes associated with coxsackievirus B3 infections but not with influenza A (23).

Indices of alveolarization and pulmonary vascular development were impaired to a greater extent in hyperoxia-exposed XYM and XYF mice compared with similarly exposed XXM and XXF mice. This led us to speculate that either Y-chromosome–specific genes were increasing susceptibility or that genes that were expressed to a higher extent in XXM and XXF mice due to incomplete X-inactivation (X-escapees) were providing protection against hyperoxic lung injury. The increased overlap in chromosomally female mice at P21, when most other genotypes diverged, points to a greater modulating effect of the female chromosomal sex on gene expression after early hyperoxia exposure.

The biological pathways that were enriched by male or female gonadal or chromosomal sex were distinct at P5 and P21. Since sex chromosomal effects were significant in the lung phenotype at P21, a closer examination of the enriched biological pathways by chromosomal sex could identify new molecular targets for pharmaco-chemical intervention. The enrichment of cell-cycle–related pathways in XX mice and immune-related pathways in XY mice at P21 may dictate the recovery response of these lungs following early hyperoxia exposure. The gene signatures in the hyperoxia-exposed murine lungs in XXF, XXM, and XYF mice were anticorrelated with the BPD signature of human female preterm babies. This was not observed with the XYM mouse lung with either the male or the female human gene expression. This further leads us to speculate that the feminized lung modulates biological pathways that protect against hyperoxia-mediated lung injury.

There are limitations to this study. The use of 95% FiO_2_ during P1–P4 (saccular stage of lung development) is high. The main reason to restrict the hyperoxia exposure to the first 4 postnatal days was to restrict the hyperoxia exposure during the saccular stage of lung development in mice (24). This corresponds to 26–36 weeks in human lung development (25). The diagnosis of BPD is made in human preterm neonates at 36 weeks of after menstrual age based on their level of respiratory support (26). For these reasons, we chose to limit the hyperoxia exposure to the saccular stage of lung development for this manuscript. Prolonging the hyperoxia exposure to 14 days would correlate with human patients receiving hyperoxia throughout their first year of life and beyond. Our previous publications (27, 28), as well as publications from other labs (29–32), have shown the long-term effects on the developing lung after hyperoxia exposure during the saccular stage of lung development. However, the use of high FiO_2_ may replicate the clinical course of babies with severe lung disease who need higher FiO_2_ and are at greatest risk to develop severe BPD. We used MLI and RAC as morphometric indices to describe the changes in the lung parenchyma. There are limitations to these indices for their use in quantitative morphology of the lung, and they have been expanded on previously (33, 34). The use of F4/80 immunostaining for quantitation of lung macrophages does not distinguish between resident alveolar and recruited macrophages and is expressed on other cells such as eosinophils (35). Whole lung RNA-Seq prevents the elucidation of cell-specific expression of DEGs. However, we attempted to speculate the cell-specific role of the selected DEGs based on the LungMAP database. Even though we showed that the chromosomal sex and not the gonadal sex had a profound effect on the injured neonatal lung during recovery, the protective effect of the double dose of the X chromosome versus the deleterious effect of the Y chromosome still needs to be clarified. Interestingly, in our study, none of the Y-chromosome genes were differentially modulated by hyperoxia exposure, leading us to speculate that the attenuation of the lung injury in the XXF and XXM mice may be mediated through genes on the X chromosome. An X-chromosome gene that escapes X-inactivation may be protective in the chromosomal female mice. Cell-specific expression of genes localized on the X chromosome will provide further insight. Species-related differences in X-inactivation also need to be taken into account. In humans, more X-chromosome–linked genes escape inactivation compared with mice (36). The correlation between human blood samples and mouse lung samples is not ideal, but these findings may point to pathways that are altered systemically (including the lung) that play a role in disease pathogenesis and vary by biological sex.

In conclusion, using the FCG model to delineate the contribution of chromosomal sex versus gonadal sex in neonatal hyperoxic lung injury should be considered an important but preliminary step toward identifying the mechanisms behind sex-specific differences in BPD. Depending on the sex chromosome complement, the effect of gonadal hormones might vary. Our future directions will focus on identifying the specific genes on the X chromosome that could be mediating the protective effect in chromosomal females and to identify their expression patterns in the lung and mechanisms of action.

## Methods

### Animals.

FCG mice were obtained from the Jackson Laboratory (stock no. 010905) and bred according to the protocol. When a WT XXF female is bread with the XYM male (both on C57BL/6J background), the pups belong to any one of the 4 genotypes (XXM, XYM, XXF, and XYF). Mice were genotyped by genomic PCR to detect the Y chromosome, *Sry*, and a control autosomal gene, as described (7).

### Mouse model of BPD.

The FCG mouse pups were exposed to hyperoxia (95% O_2_), as described previously, to replicate the arrest of lung development as seen in human neonates with BPD (3, 37). Pups were euthanized on P5 (immediately after hyperoxia exposure) or P21 (after recovery in room air; during alveolar phase of lung development) (Figure 1). Evaluation of lung morphometry (MLI and RAC), lung vascular development (IHC for vWF), and lung macrophage quantitation (IHC for F4/80) was performed at P21 as previously described (3). The detailed protocol is included in Data Supplement.

### Analysis of the whole lung transcriptome.

RNA-Seq analysis was performed on the whole lung mRNA to analyze differences in the pulmonary transcriptome among the FCG mice at the 2 time points: P5 (end of saccular stage of lung development) and P21 (alveolar stage of lung development), as shown in Figure 1. Sex of the animals was ascertained using genotyping for the *Sry* (sex-determining region Y) gene. Figure 1 also shows the RNA-Seq signal (defined as FPKM) for *Uty* (a Y-chromosome–specific gene), *Xist* (an X-chromosome–specific gene expressed only when more than 1 X chromosome is present), and *Sry* in representative samples for each of the experimental groups submitted for RNA-Seq analysis. The detailed methodology for the RNA-Seq analysis is included in the Data Supplement. The data set has been deposited in NCBI GEO (GSE162853).

### Validation of RNA-Seq data.

qPCR analysis was conducted on lung mRNA from an independent cohort of FCG neonatal mice to validate the results of the RNA-Seq analysis. The genes chosen and the primer information is included in the Data Supplement. The expression of these genes at the cellular level was predicted using the LungMAP database (38, 39).

### Human transcriptomic data mining.

Transcriptomes for healthy adult whole lung from the GTEx consortium were downloaded from the GTEx data portal. Blood transcriptome data from newborns was downloaded from NCBI GEO (accession no. GSE32472) (15). The human transcriptome data mining and analysis is described in detail in the Data Supplement.

### Statistics.

Data analysis for lung morphometry, pulmonary vascular development, and gene expression validation was done using GraphPad Prism, version 9. Three-way ANOVA was used to assess the effect of gonadal sex, chromosomal sex, and treatment, as well as the interaction between the 3 independent variables. Comparison between individual groups was performed using Sidak’s multiple-comparison test. The number of biological replicates is specified for each experiment. *P* < 0.05 was considered statistically significant.

### Study approval.

All studies were conducted at Baylor College of Medicine under the purview of the IACUC (protocol no. AN-6474). Experiments were performed in accordance with the highest contemporary standards as per the IACUC protocols.

## Author contributions

Conception of projects and study design was contributed by KL. Conducting of experiments, data analysis, and interpretation were contributed by KL, CC, SLG, and APA. XD, YZ, and AFC contributed in the conduct of experiments, data analysis, and interpretation. BM contributed to data analysis, interpretation, and writing and editing of the manuscupt. All authors contributed to drafting the manuscript and intellectual contribution.

## Supplementary Material

Supplemental data

Supplemental Table 1

Supplemental Table 2

Supplemental Table 3

## Figures and Tables

**Table 1:**
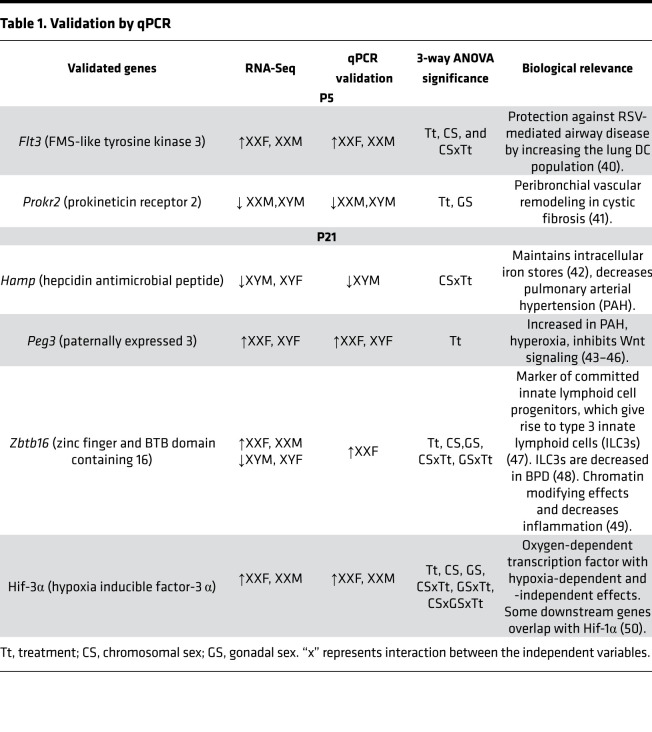
Validation by qPCR

## Version 1 06/01/2021

In-Press Preview

## Version 2 07/08/2021

Electronic publication
