# Supplementary material for: Effect of sex chromosomes versus hormones in neonatal lung injury
Source: JCI Insight. 2021 Jul 8;6(13):e146863. doi: 10.1172/jci.insight.146863 (PMC8410054; doi:10.1172/jci.insight.146863)
Supplement: Supplemental data [file jciinsight-6-146863-s134.pdf]

**Online Data Supplement**

# Effect of sex chromosomes versus hormones in neonatal hyperoxic lung injury

Sandra L. Grimm, Xiaoyu Dong, Yuhao Zhang, Alex Carisey, Arthur P. Arnold, Bhagavatula Moorthy, Cristian Coarfa, Krithika Lingappan

**Model of BPD:** Pups from multiple litters were pooled (litter size limited to 6 per dam) before being randomly redistributed to the normoxia (21% $O_2$) and the hyperoxia (95% $O_2$) group, within 12 h of birth for 4 days (saccular stage of lung development; equivalent to 26-36 weeks in human gestation). Every 24 hours, the dams were swapped between air- and hyperoxia-exposed litters to prevent toxicity due to hyperoxia exposure and to eliminate maternal effects between the groups.

**Lung Histology and Morphometry:** Mice were anesthetized (100 mg/kg sodium pentobarbital i.p.) and lungs were fixed with 4% paraformaldehyde endotracheally at 25 cm $H_2O$ pressure for 15 minutes. Lungs were further fixed overnight at 4°C followed by dehydration in graded alcohol and embedded in paraffin. Lung sections (5 um thick) were prepared, stained with hematoxylin-eosin and radial alveolar counts (RAC) and mean linear intercept (MLI) was measured as described before. MLI analysis using measurements from all the five lung lobes. Coronal lung sections were used cut at a plane close to the hilum of the lung. This sectioning was consistent among all mice. Each lung had at least more than 20 regions of interest (ROI) evaluated in the distal lung (excluding areas with major vessels or airways). All possible regions of lung were included in the analysis. Furthermore, we used a quantitative semi-automated measurement of mean linear intercept (1). Both horizontal and vertical test lines were used to measure horizontal and vertical intercepts in each ROI. This led to well over 15000 measurements in each biological replicate (2). The anonymized slides were imaged using a Zeiss Imager M.2 (Zeiss Microscopy) fitted with a Lumenera Infinity3 6.0 MP CCD Color camera (Teledyne) and a LEP BioPoint2 motorized stage (Ludl) controlled by μManager (3)(v. 1.4). Individual fields of view were corrected for uneven illumination and

tiled together to recreate a seamless image of the entire tissue section using Aivia 9.8 (Leica Microsystems). Using Fiji (v. 1.53h), individual regions of interest of 675 μm x 675 μm were drawn to cover as much as possible of the lung parenchyma across all 5 lobes, while avoiding major vessels area and airways. The individual regions of interest were subsequently batch processed using MLI plugin for ImageJ/Fiji (4) to measure the mean linear intercept distance along each line, in each ROI, and each tissue section. The spacing between each line (both horizontal and vertical) was set to 15 pixels (or 11.25 μm) after determination that this was roughly equivalent to the radius of a single airways, hence guaranteeing an ideal sampling rate for the MLI measurements. Raw results were then analyzed using a custom-made R script (R Studio Desktop, v. 1.4.1106) and finally de-anonymized in order to assemble animal repeats and conditions prior to final plotting using GraphPad Prism (v. 8.4.3, GraphPad Software).

**Immunohistochemistry:** Pulmonary vessel density was determined based on vWF (1:4000 dilution, abcam; cat no ab6994) immunostaining. vWF-stained vessels with external diameter less than 50 μm per high-power field were counted in 10 random non-overlapping fields (x200 magnification; n=6/group). Care was taken to avoid fields containing large airways or vessels. F4/80 antibody for macrophage (1:500 dilution, Bio Rad laboratories; catalog number:MCA497G) was used for staining the lung macrophages. Twenty random non-overlapping high-power fields were analyzed and numbers of cells were counted and the average number of cells per high power field was calculated.

**RNA isolation:** Total RNA from flash frozen lung samples was isolated using the ZYMO mRNA extraction kit. Sample concentration and quality was assayed using Nanodrop-8000

(Thermo Scientific, Wilmington, DE, USA) and further with the Agilent Bioanalyzer. The 260/280 and 260/230 ratios were greater than 1.8. and the RNA Integrity Number (RIN) were between 7-10 and with a range of 1 – 1.5.

**RNA-Sequencing Library Preparation and Sequencing.**

RNA samples underwent quality control assessment using the RNA tape on Tapestation 4200 (Agilent) and were quantified with Qubit Fluorometer (Thermo Fisher). The RNA libraries were prepared and sequenced at the University of Houston Seq-N-Edit Core per standard protocols. RNA libraries were prepared with QIAseq Stranded Total RNA library Kit (Qiagen) using 1000 ng input RNA. mRNA was enriched with Oligo-dT probes attached to Pure mRNA beads (Qiagen). RNA was enzymatically fragmented for 15 minutes, reverse transcribed into cDNA, and ligated with Illumina sequencing adaptors. The size selection for libraries was performed using SPRIselect beads (Beckman Coulter) and purity of the libraries was analyzed using the DNA 1000 tape Tapestation 4200 (Agilent). The indexed libraries were pooled and sequenced

**Lung transcriptome data analysis:** Data was trimmed using trimGalore, it was mapped using HISAT2 (5) to the UCSC mouse build mm10 and gene expression was quantified using featureCounts (6) and the GENCODE gene model (7). Data was further normalized using the RUV method, and differential gene analysis was assessed via the EdgeR method (8), using the R statistical system. We used the significance threshold of FDR-adjusted p-value<0.05, and fold change exceeding 1.5x. Enriched pathways were assessed via the over-representation method (as implemented by MsigDB) using the hypergeometric distribution

and the Gene Ontology Biological Processes compendium, with significance achieved at FDR-adjusted p-value<0.05. Signature correlation across the GTEx healthy lung samples was achieved by first considering genes conserved between mouse and human. Next, genes were converted to z-score across the human samples. Finally, the summed z-scores for a signature and human specimen was computed by adding the z-score of up-regulated genes and subtracting the z-scores of down-regulated genes. Pearson Correlation Coefficient was computed between signatures using the Python scientific library, with significance achieved at p<0.05. Pathway enrichment using Gene Set Enrichment Analysis was also assessed, with significance achieved at FDR<0.25.

**Comparison of X-chromosome and autosomal gene expression in the lung:** Feature counts for protein coding genes were normalized across all genotypes, exposures, and time points using the Trimmed Mean of M-values (TMM) and converted to counts per million (cpm). The average cpm was calculate for each group (male gonad, female gonad, male chromosome, female chromosome) at each time point (PND5 or PND21) and for each exposure (room air or hyperoxia). A ratio of the average gene expression was calculated and converted to log2 values, as previously reported (9). X and Y chromosome genes were identified based on the GENCODE gene model, and all other expressed genes were considered autosomal. Log2 ratios of autosomal and X chromosome genes were graphed in GraphPad Prism v9 using a frequency distribution analysis, with relative frequency plotted on the y-axis against log2 values with bins of 0.1 units on the x-axis.

**qRT-PCR analysis:** Total RNA was extracted from the lung tissue using Direct-zol mRNA microprep kit (ZYMO Research, R2062). cDNA was prepared using RevertAid Reverse Transcriptase kit (ThermoFisher, k1691). Quantitative PCR was performed using the QuantStudio 7 Flex real-time PCR detection system (ThermoFisher) and SYBR Green (Bio-Rad, 1725275). The thermal cycling conditions used were as follows: one cycle at 95 °C for 1 min, 40 cycles at 95 °C for 15 s, and one cycle at 60 °C for 15 s. The primers used in real-time PCR test are listed in Supplemental Table 1 Relative mRNA levels were calculated using the $2^{-\Delta\Delta CT}$ method and normalized to *Tbp* expression, which was used as the housekeeping gene.

| Gene | Forward (5'-3') | Reverse (5'-3') |
|---|---|---|
| *Flt3* | CCTGGAGTTCAAGTCGTGTG | TAGCTGGAGTCGCTCAGGAT |
| *Peg3* | GAGAACTGCCTACCCAAGCA | CGAGCCACATCCTTGATGA |
| *Hamp* | CAACTTCCCCATCTGCATCT | GGGGAGGGCAGGAATAAATA |
| *Prokr2* | GCCGTTCTATGGCTTTACCA | CTTGACCGTCACGAAGCATA |
| *Hif3-alpha* | GCTCAGAGGACAAAGGGTTG | GTCGACCTTGCAGAAGGAAA |
| *Zbtb16* | CACCAGTTGGAGACGCACTA | CACCGTTGTGTGTTCTCAGG |
| *Alox15* | CTTCCTTCTGGATGGGATCA | GGTGGGGTAGACCCAGTTTT |
| *Hk3* | GCTGTATCCTCCTGGACCAC | CACAGCCACGCACACATATT |
| *Rsad2* | TCAAAAGCTGAGGAGGTGGT | AGGAGGCACTGGAAAACCTT |
| *Ltf* | ACCTAGCCATAGCCCCAAAC | CAGGCAAAACTCTCCTGGAC |
| *Nr1d1* | TCGGGAGGTGGTAGAGTTTG | TCACTGTCTGGTCCTTCACG |
| *Tlr7* | CTTGCCTCCTGAGGTTTTTG | GCTGAGGTCCAAAATTTCCA |
| *Tbp* | GGACCAGAACAACAGCCTTC | CCGTAAGGCATCATTGGACT |

**Human transcriptome data mining and analysis:**

When comparing and contrasting gene signatures (differentially expressed genes, DEGs), it is important to account for the fact that in real tissues there are complex underlying

relationships between genes, such as genes in the same pathway or genes at different locations in a signaling cascade of distinct pathways. Also, whereas some housekeeping genes are fairly constant across individuals, other genes exhibit high variability in a population. To capture and quantify the complex underlying dependencies between genes in one individual tissue and variability between individuals, cancer bioinformatics has developed the summed z-scores methodology, applied over the cohort of whole tissue transcriptomes (10–12). Data for 578 healthy whole lung transcriptomes from human donors was downloaded from the GTEx consortium (13). Genes were next transformed to a z-score across all samples. For each gene signature and each specimen, we obtained the summed z-scores by adding the z-scores of up-regulated genes and subtracting the z-scores of down-regulated genes, via a methodology used extensively in mining of transcriptomic cohorts from cancer patients (11, 14). Gene symbols were converted to mouse genome, using the Ensemble BiomaRt reference (15). Essentially, the summed z-score compares each human specimen with the average gene expression in the human cohort across all the Four Core Genotype (FCG) signatures, and then scores each human specimen based on how well it matches each FCG DEG list. With each human specimen in a cohort assigned a summed z-score, it is possible then to do a Pearson Correlation Coefficient analysis. Significant correlations between distinct FCG signatures can then indicate underlying biological relationships. Signature correlations in transcriptomics from GTEx lungs were evaluated using Pearson correlation, with significance achieved at $p<0.05$.

Data for blood transcriptomes from newborns was downloaded from NCBI GEO, accession number GSE32472(17). In this cohort, babies were enrolled if gestational age was < 32

weeks and birth weight less than or equal to 1500 gms. The data was collected from 111 preterm neonates with a mean birth weight of 1029 g and mean gestational age at birth of 27.8 weeks. BPD was diagnosed in 68 (61%) infants (40 with mild disease, 13 with moderate and 15 with severe BPD). Forty-three newborns served as a control group (no BPD). Blood was collected from the participants postnatally and the latest time-point was at 28 days of life after birth, for the assessment of whole genome expression in peripheral blood leukocytes. BPD status was encoded as 0 for control, 1 for mild BPD, 2 for moderate BPD, and 3 for severe BPD. Oxygen need at 28 days was encoded as 0 for no therapy and 1 for therapy. Association with clinical variables such as birth weight, gestational age, BPD, or oxygen treatment at 28 days was assessed using the Pearson correlation test, with significance achieved for $p<0.05$. Results were plotted using GraphPad prism. Signatures of BPD status in the GSE32472 (16) cohort were assessed via a ttest parametric testing, with significance achieved for FDR-adjusted p-value<0.05. Gene set enrichment analysis was carried out using the GSEA software.

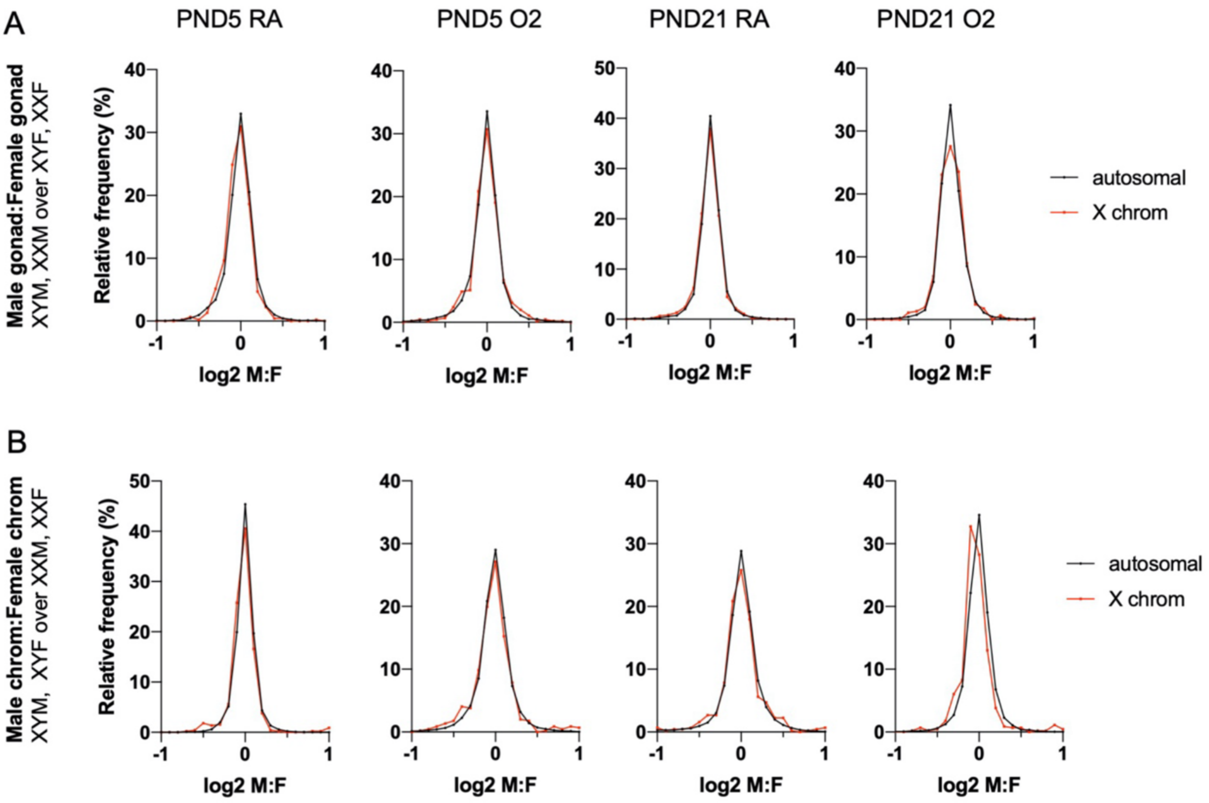


**Supplemental Figure 1: Frequency distribution of male-to-female $log_2$ratios of gene expression comparing distributions across all expressed genes between the X chromosome and autosomes. A.** The frequency distribution of $log_2$ of male:female ratios (expressed as a percentage), is plotted for autosomal (black, n=13,455) or X chromosome (red, n=446) genes when comparing lung gene expression from mice with male gonads (XYM, XXM) to those with female gonads (XXF, XYF). Graphs for each timepoint (PND5 or PND21) and each condition (RA or O2) are shown. A $log_2$ ratio of 0 is equal to a 1:1 M:F ratio of gene expression. **B.** Frequency distribution of $log_2$ male:female ratios is plotted for chromosome X and autosomal genes, comparing gene expression from mice with male chromosomes (XYM, XYF) to those with female chromosomes (XXF, XXM).

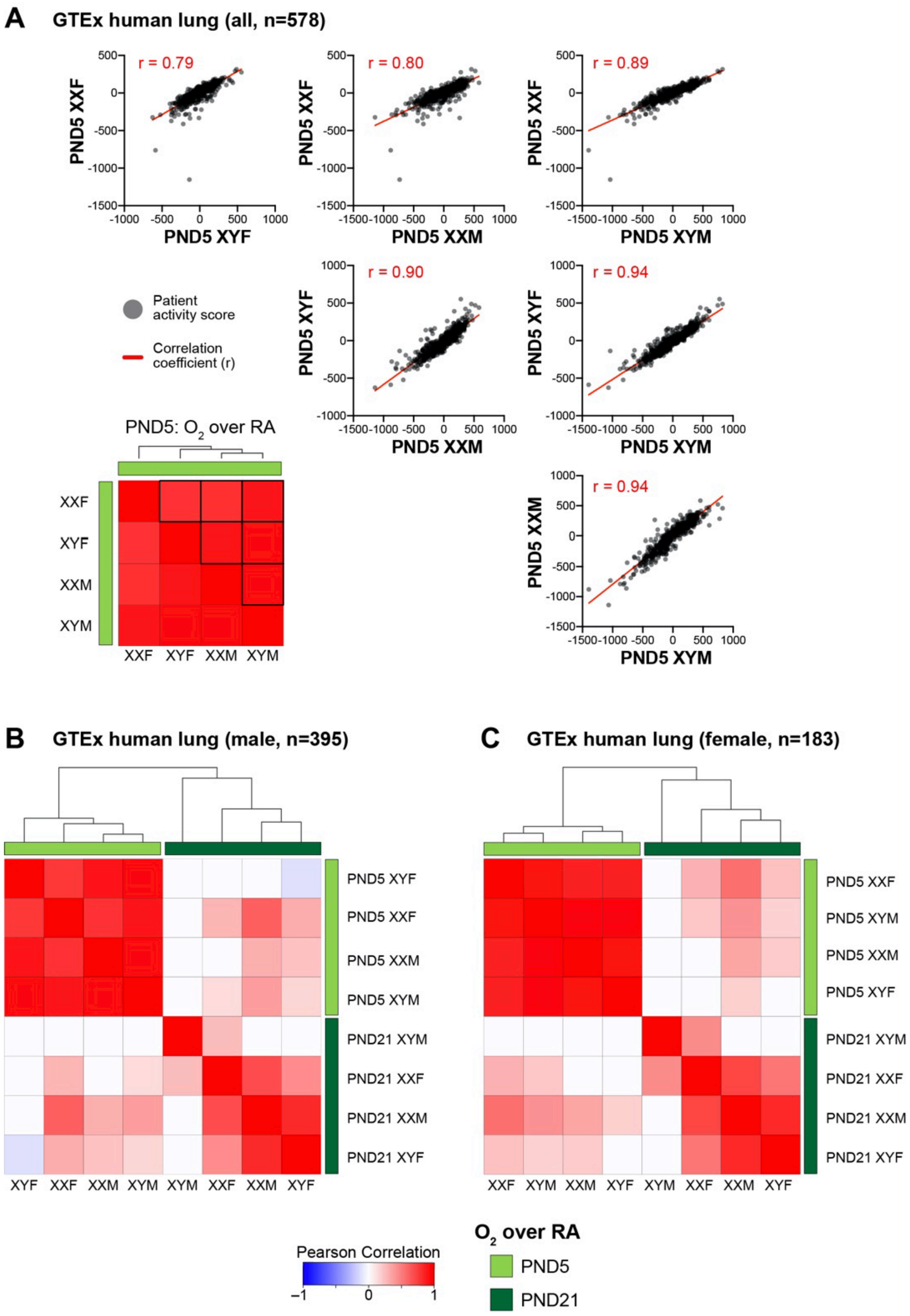

A
GTEx human lung (all, n=578)
r = 0.79
r = 0.80
r = 0.89
r = 0.90
r = 0.94
r = 0.94
PND5 XXF
PND5 XYF
PND5 XXM
PND5 XYM
Patient activity score
Correlation coefficient (r)
PND5: $O_2$ over RA
XXF
XYF
XXM
XYM
B
GTEx human lung (male, n=395)
PND5 XYF
PND5 XXF
PND5 XXM
PND5 XYM
PND21 XYM
PND21 XXF
PND21 XXM
PND21 XYF
C
GTEx human lung (female, n=183)
PND5 XXF
PND5 XYM
PND5 XXM
PND5 XYF
PND21 XYM
PND21 XXF
PND21 XXM
PND21 XYF
Pearson Correlation
−1
0
1
$O_2$ over RA
PND5
PND21

**Supplemental Figure 2: Distribution of summed z-score for hyperoxia responses show striking patterns in healthy adult lungs.** Scatterplots supporting the strong correlation of summed z-scores for PND5 hyperoxia exposure responses over 578 human adult lungs are presented, indicating that hyperoxia responses are very similar at the early phase between the four genotypes in the FCG model (**Figure 2A**). Summed z-scores correlations for PND5 hyperoxia exposure responses over 395 human adult male (**Figure 2B**) and 183 adult female lungs (**Figure 2C**) indicate strong correlations of the acute response.

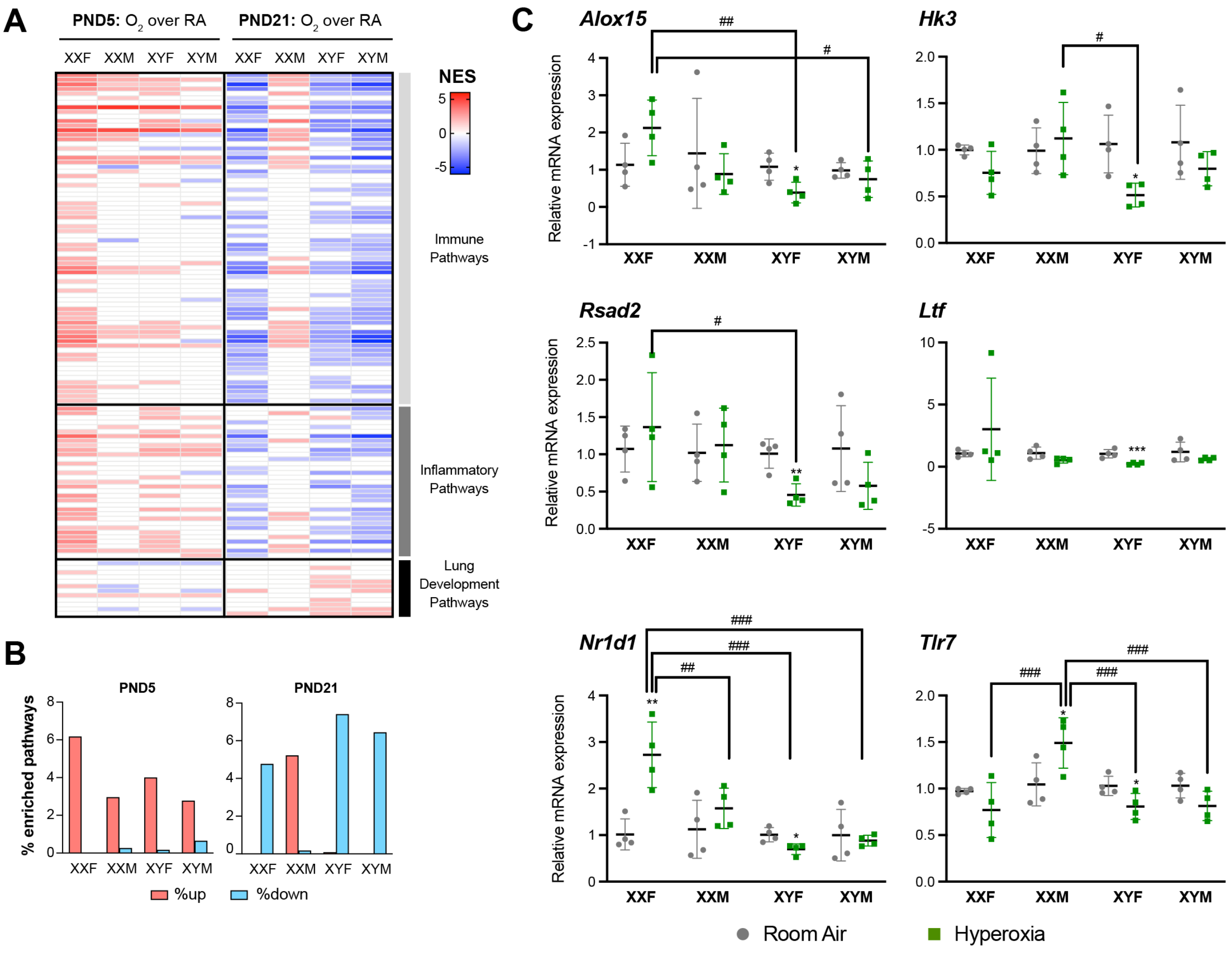


**Supplemental Figure 3: Distribution of enriched biological pathways related to immune/inflammation and lung development based on the gonadal or chromosomal sex in the FCG mouse model at PND5 and PND21 after hyperoxia exposure.** Heatmap of normalized enrichment scores (NES) of the enriched pathways related to immune, inflammation and lung development in FCG mice exposed to hyperoxia at PND5 and PND21 (Figure 3A). Percent of enriched GOBP pathways (upregulated and downregulated) that are immune or inflammation related in the FCG mice exposed to neonatal hyperoxia at PND5 and PND21 (Figure 3B). The fold change of selected genes related to immune/inflammation related pathways in the RNA-Seq experiment was validated in an independent cohort of neonatal mice by qRT-PCR at PND21 (**Figure 3C).** Values are mean ± SD from 4 individual

animals. Analysis done by 3-way ANOVA to assess the effect of treatment, chromosomal sex and gonadal sex as well as the interactions between the independent variables. Significant differences between room air and hyperoxia within genotype are indicated by *$P<0.05$, **$P<0.01$, and ***$P<0.001$. Significant differences between hyperoxia-exposed mice between different genotypes are indicated by $^{\#}P < 0.05$, $^{\#\#}P < 0.01$, and $^{\#\#\#}P < 0.001$.

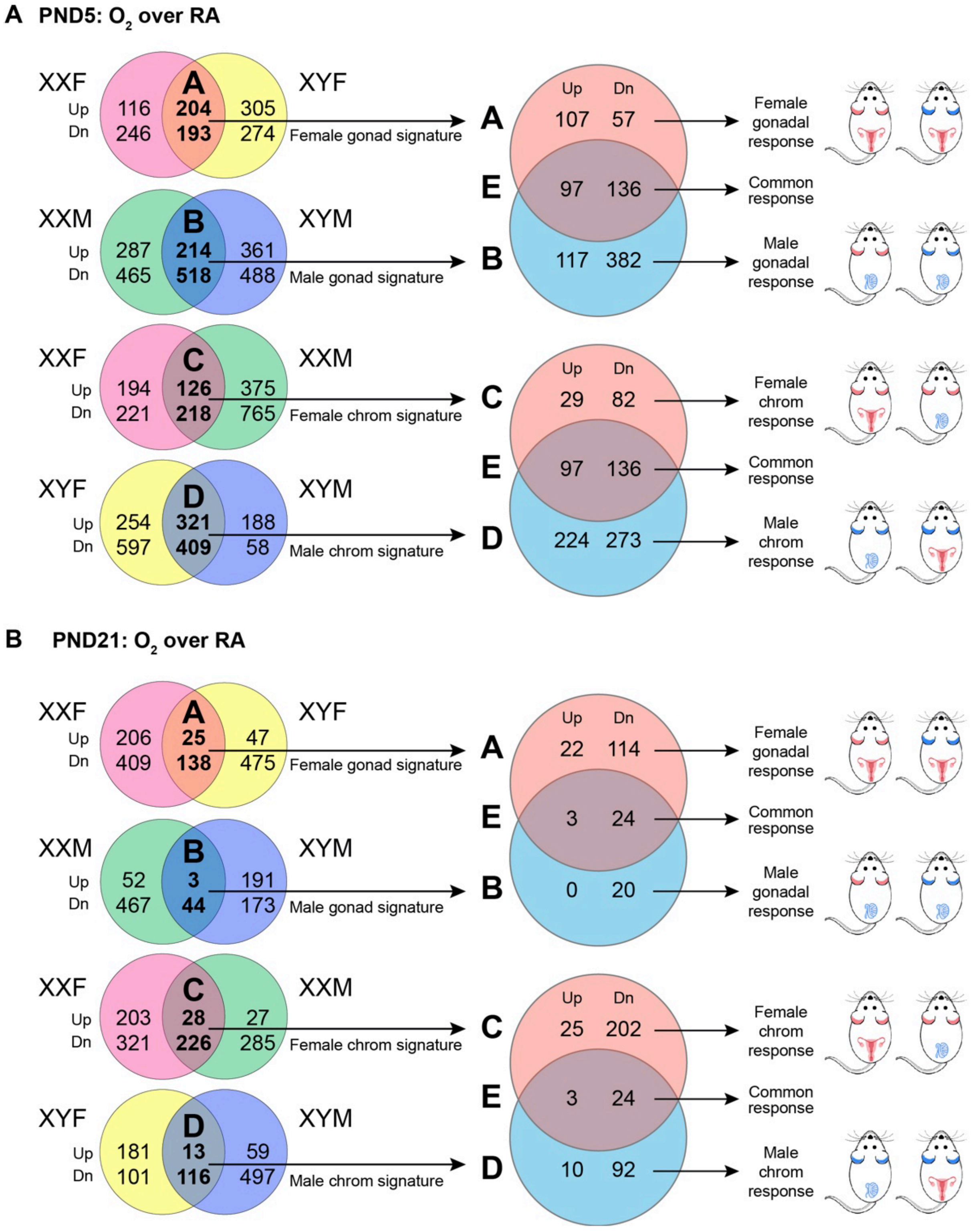

A PND5: $O_2$ over RA
XXF
XYF
Up
Dn
116
246
A
204
193
305
274
Female gonad signature
XXM
XYM
Up
Dn
287
465
B
214
518
361
488
Male gonad signature
XXF
XXM
Up
Dn
194
221
C
126
218
375
765
Female chrom signature
XYF
XYM
Up
Dn
254
597
D
321
409
188
58
Male chrom signature
Up Dn
A 107 57
E 97 136
B 117 382
Up Dn
C 29 82
E 97 136
D 224 273
Female gonadal response
Common response
Male gonadal response
Female chrom response
Common response
Male chrom response
B PND21: $O_2$ over RA
XXF
XYF
Up
Dn
206
409
A
25
138
47
475
Female gonad signature
XXM
XYM
Up
Dn
52
467
B
3
44
191
173
Male gonad signature
XXF
XXM
Up
Dn
203
321
C
28
226
27
285
Female chrom signature
XYF
XYM
Up
Dn
181
101
D
13
116
59
497
Male chrom signature
Up Dn
A 22 114
E 3 24
B 0 20
Up Dn
C 25 202
E 3 24
D 10 92
Female gonadal response
Common response
Male gonadal response
Female chrom response
Common response
Male chrom response

**Supplemental Figure 4: Distribution and overlap of gene signatures based on the gonadal and chromosomal sex: Figure 4** shows the schematic and the number of differentially expressed genes (up- and down-regulated) in each genotype and the overlap based on gonadal or chromosomal sex. **Figure 4A** shows the distribution at PND5 and **Figure4B** at PND21.

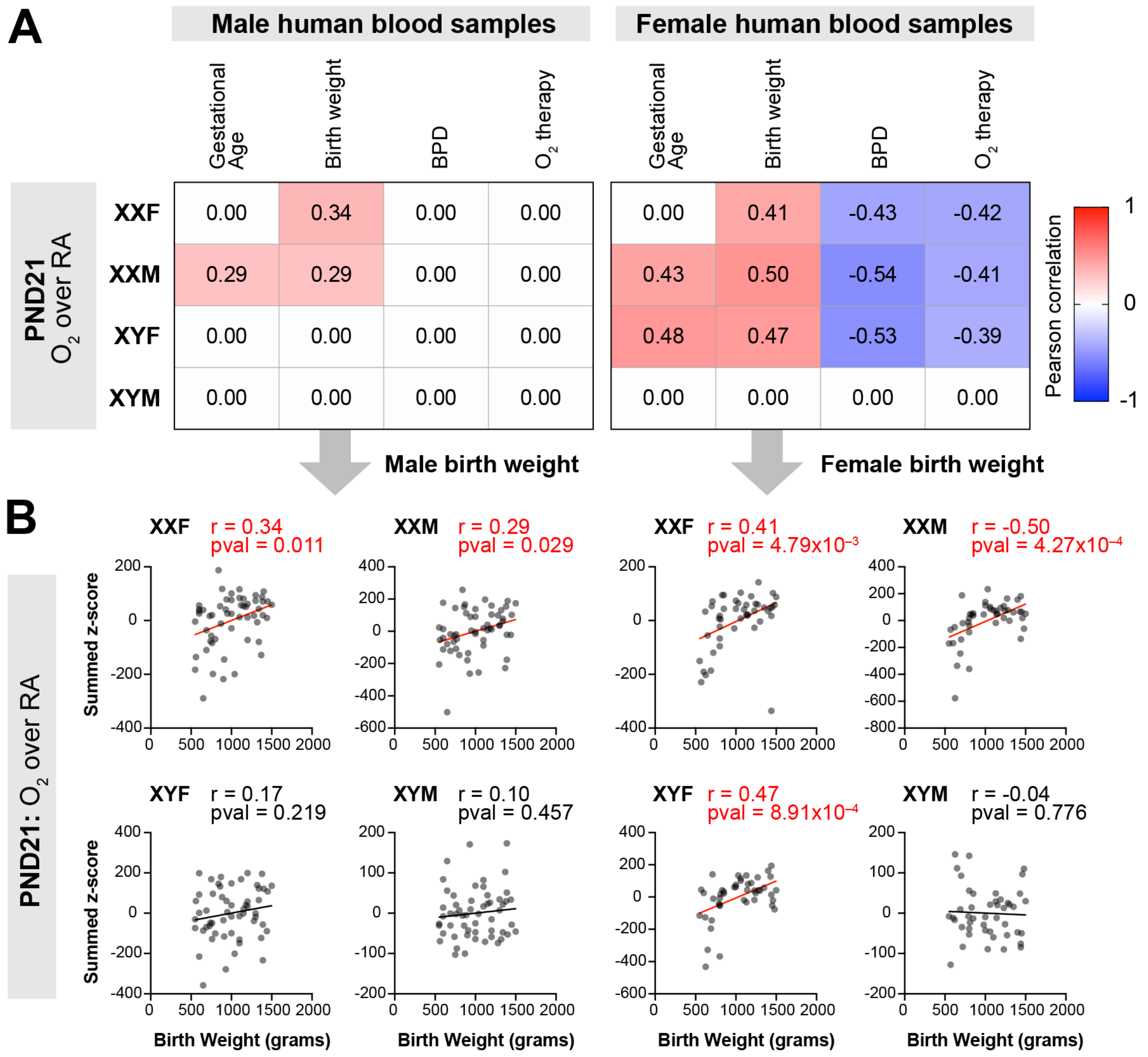


**Supplemental Figure 5: Association between summed z-scores for hyperoxia DEGs and BPD clinical variables in a human newborn cohort.** Association between summed z-scores for hyperoxia DEGs at PND21 and four clinical variables: gestational age, birth weight, BPD status and whether oxygen requirement at 28 days postnatal age are presented. The Pearson correlation coefficient is shown for significant correlation (p<0.05). This information is also presented in Figure 11A and reproduced here for the reader's convenience (**Figure 5A**). Scatterplots indicating the correlation between activity scores of hyperoxia signatures at PND21 and birth weights patients from a BPD cohort (**Figure 5B**).

Interestingly, there was a positive correlation between the patient activity scores of chromosomal female mice and birth weight in the male and female patient cohort.

Table 1: Validation of immune/inflammation pathway related genes by qRT-PCR

| Validated genes | RNA-Seq | qRT-PCR validation | 3-way ANOVA significance[A] | Biological relevance |
|---|---|---|---|---|
| *Alox15* (Arachidonate 15-Lipoxygenase) | ↓ XYF, XYM | ↓ XYF | CS | Expressed in myeloid cells (resident and alternatively activated macrophages). Plays a role in resolution of inflammation and dendritic cell maturation (17, 18). |
| *Hk3* (Hexokinase 3) | ↓ XYF, XYM | ↓ XYF | Tt | Catalyzes the first step in glucose metabolism. Protective against oxidative stress(19). Functions as a metabolic switch for cell survival during myeloid differentiation (20). |
| *Rsad2* (Radical S-Adenosyl Methionine Domain Containing 2) | ↓ XYF, XYM | ↓ XYF | CS, Tt | Interferon-stimulated gene involved in innate immunity. Necessary for dendritic cell maturation (21). |
| *Ltf* (Lactotransferrin) | ↑ XXF<br>↓ XXM,XYF, XYM | ↓ XYF | - | Host-defense peptide. May play a role in the generation of myeloid derived suppressor cells in the neonate (22) |
| *Nr1d1* (Nuclear Receptor Subfamily 1 Group D Member 1) | ↑ XXF<br>↓XXM,XYM, XYF | ↑ XXF, ↓ XYF | Tt, CS, CSxTt, GSxTtxCS | Coordinates circadian rhythm and metabolic pathways. Plays a role in the suppression of the endogenous proinflammatory mechanism in the lung (23). |

| | | | | |
|---|---|---|---|---|
| | | | | |
| *Tlr7 (Toll like receptor 7)* | ↓ XYF, XYM | ↑ XXM, ↓ XYF | CS, GS, CSxTt, GSxTt, CSxGSxTt | Present on the X chromosome. Escapes C-chromosome inactivation in immune cells (24). |
| [A] Tt: treatment; CS: chromosomal sex; GS: gonadal sex<br>"x" represents interaction between the independent variables | | | | |

**Supplemental References:**

1. Crowley G et al. Quantitative lung morphology: Semi-automated measurement of mean linear intercept. *BMC Pulm Med.* 2019;19(1):1–9.

2. Mitzner W. Use of mean airspace chord length to assess emphysema. *J Appl Physiol.* 2008;105(6):1980-1981.

3. Edelstein AD et al. Advanced methods of microscope control using μManager software. *J Biol Methods*. 2014;1(2):e10.

4. Schindelin J et al. Fiji: An open-source platform for biological-image analysis. *Nat. Methods*. 2012;9(7):676-682.

5. Kim D, Langmead B, Salzberg SL. HISAT: A fast spliced aligner with low memory requirements. *Nat. Methods*.2015;12(4):357-360.

6. Liao Y, Smyth GK, Shi W. FeatureCounts: An efficient general purpose program for assigning sequence reads to genomic features. *Bioinformatics.* 2014;30(7):923-930.

7. Harrow J et al. GENCODE: producing a reference annotation for ENCODE. *Genome Biol.* 2006;7 Suppl 1(Suppl 1):S4.1-9.

8. Robinson MD, McCarthy DJ, Smyth GK. edgeR: a Bioconductor package for differential expression analysis of digital gene expression data. *Bioinformatics.* 2010;26(1):139–140.

9. Itoh Y et al. Dosage compensation is less effective in birds than in mammals. *J Biol.* 2007;6(1):2.

10. Taylor BS et al. Integrative Genomic Profiling of Human Prostate Cancer. *Cancer Cell*. 2010;18(1):11-22.

11. He B et al. GATA2 facilitates steroid receptor coactivator recruitment to the androgen receptor complex. *Proc Natl Acad Sci U S A.* 2014;111(51):18261-18266.

12. Geng C et al. SPOP regulates prostate epithelial cell proliferation and promotes ubiquitination and turnover of c-MYC oncoprotein. *Oncogene*. 2017;36(33):4767-4777.

13. Aguet F et al. Genetic effects on gene expression across human tissues. *Nature*. 2017;550(7675):204-213.

14. Vantaku V et al. Multi-omics integration analysis robustly predicts high-grade patient survival and identifies CPT1B effect on fatty acid metabolism in Bladder Cancer. *Clin. Cancer Res.* 2019;25(12):3689-3701.

15. Durinck S, Spellman PT, Birney E, Huber W. Mapping identifiers for the integration of genomic datasets with the R/ Bioconductor package biomaRt. *Nat Protoc.* 2009;4(8):1184-1191.

16. Pietrzyk JJ et al. Gene Expression Profiling in Preterm Infants: New Aspects of Bronchopulmonary Dysplasia Development. *PLoS One* 2013;8(10):e78585.

17. Rothe T et al. 12/15-lipoxygenase-mediated enzymatic lipid oxidation regulates DC maturation and function. *J Clin Invest.* 2015;125(5):1944-1954.

18. Sica A, Mantovani A. Macrophage plasticity and polarization: In vivo veritas. *J Clin Invest.* 2012;122(3):787-795.

19. Wyatt E et al. Regulation and cytoprotective role of hexokinase III. *PLoS One*. 2010;5(11):e13823.

20. Seiler K et al. Hexokinase Proteins Impart Distinct Functions in Myeloid Development and Cell Death. *Blood*. 2018;132(Supplement 1):5088.

21. Jang JS et al. Rsad2 is necessary for mouse dendritic cell maturation via the IRF7-mediated signaling pathway. *Cell Death Dis.* 2018; 9(8):823.

22. Liu Y et al. Lactoferrin-induced myeloid-derived suppressor cell therapy attenuates

pathologic inflammatory conditions in newborn mice. *J Clin Invest.* 2019;129(10):4261-4275.

23. Pariollaud M et al. Circadian clock component REV-ERBa controls homeostatic regulation of pulmonary inflammation. *J Clin Invest.* 2018;128(6):2281-2296.

24. Souyris M et al. TLR7 escapes X chromosome inactivation in immune cells. *Sci Immunol.* 2018;3(19):eaap8855.
